# The Diagnostic Challenge of an Infrequent Spectrum of* Cryptococcus* Infection

**DOI:** 10.1155/2019/5970648

**Published:** 2019-01-02

**Authors:** Francisco Barbosa De Araujo Neto, Camila Corona De Godoy Bueno, Liege Tambelini Gomes, Daniela Alejandra Ortiz Navas, Mark Wanderley, Stefanie Gallotti Borges Carneiro, Rita Karine Veras Gomes De Mello, Laura Mendes Coura, Larissa Sayuri Missumi, Henrique Durante, Ricardo Francisco Cintra Zagatti, Márcio Valente Yamada Sawamura

**Affiliations:** ^1^MD, Radiologist, Department of Radiology of the Medical School of the University of São Paulo, São Paulo, SP, Brazil; ^2^MD, Doctor Resident in Radiology, Department of Radiology of the Medical School of the University of São Paulo, São Paulo, SP, Brazil; ^3^MD, Pathology Resident, Department of Pathology of the Medical School of the University of São Paulo, São Paulo, SP, Brazil; ^4^MD, Radiologist, Department of Radiology of the Hospital Heliópolis, São Paulo, SP, Brazil

## Abstract

Cryptococcal infection results from inhalation of fungal spores and usually is confined to the lungs, but may disseminate systemically. Radiologically, cryptococcal infection has multiple forms of presentation. The diagnosis is usually based on fungal isolation from cultured clinical specimens. Long term antifungal therapy is recommended, but surgical procedures may eventually be necessary when large thoracic symptomatic masses are present. We report a case of a 41-year-old male, immunocompetent, investigating a palpable mass in the left supraclavicular region associated with unintentional weight loss over the last three months. He also reported chest pain in this period. Chest X-ray, ultrasonography, and computed tomography were performed, which diagnosed a mediastinal and left supraclavicular mass, interpreted as lymph node conglomerates of unknown etiology. He also underwent a biopsy of the left supraclavicular mass for etiological determination by histopathology, which confirmed cryptococcosis infection. Although very infrequent, mediastinal cryptococcal infection (simulating masses) is a challenging but important differential diagnosis of benign and malignant lesions, since its treatment is usually clinical.

## 1. Introduction

Cryptococcal infection usually results from inhalation of fungal spores and may be confined to the lungs or disseminate systemically. The possible imaging findings of pulmonary cryptococcosis are single well-defined consolidation or mass, diffusely scattered pulmonary nodules, or interstitial opacities [1–4]. In immunocompetent patients, the infection usually is confined to the lungs, but in immunocompromised patients it may spread, typically to central nervous system (CNS). Cryptococcal lymphadenopathy, especially affecting the mediastinum, is mainly reported in patients infected with the human immunodeficiency virus (HIV) [5–9]. In cryptococcal lymphadenitis, lymph nodes are generally <1.5 cm and accompanied by pulmonary parenchymal changes [10].

The diagnosis of cryptococcosis is usually based on isolation of the fungus from cultured clinical specimens, but it requires several days and a large amount of samples. The detection of cryptococcal capsular antigen in pulmonary and cerebrospinal fluid (CSF) specimens by pulmonary agglutination is one of the most helpful adjunct techniques to diagnose cryptococcosis because of its good sensitivity [1–7].

The Infectious Diseases Society of America published a clinical practice guideline for the management of cryptococcal disease [11–15]. It recommends amphotericin B with flucytosine followed by fluconazole for cryptococcal meningoencephalitis and severe pulmonary cryptococcosis in immunocompetent patients [11–15]. Recent studies have reported that eculizumab may have an antagonistic effect in the treatment of cryptococcal infection spectra, with studies and research indicating that by a hypothetical change in immunomediation [16].

Surgery should be considered for either diagnosis or persistent radiographic abnormalities and symptoms not responding to antifungal therapy. Compression of vital structures, failure to reduce the size of the cryptococcoma after four weeks of therapy, and failure to thrive are also surgery indications.

## 2. Case Report

We report a case of a 41-year-old male, immunocompetent, with no other comorbidities who went to the hospital to investigate unintentional weight loss in the last three months and to investigate a hard and palpable mass in the left supraclavicular region. He underwent series of laboratory tests and several imaging tests, such as blood cells count, T-cells immunophenotypes, analysis of B and NK-cells, and expression of interferon gamma receptor searching for immunodeficiencies—all in the normal range: neutrophils 5,72 mil/mm3 (reference titles 4,00 -11,00 mil/mm3), lymphocytes 3,69 mil/mm3 (reference titles 1,60 - 7,00 mil/mm3), monocytes 0,55 mil/mm3 (reference titles $\underline{0\text{,}20\;\text{-}\;0\text{,}90\;mil/mm3)\text{,}}$ and eosinophils 0,07 mil/mm3 (reference titles 0,05 - 0,50 mil/mm3); inflammatories parameters like C-reactive protein 151,1 mg/L (reference titles < 5,0 mg/L) were elevated; and inflammatories parameters like DHL 191 mg/L (reference titles 135-225 mg/L) were normal. Cryptococcal capsular antigen dosages were made in the blood and spinal fluid, giving positive results (reagents) with a titre of 1:32. Viral serologies like HIV, hepatitis, and HTLV were all negative; acid-alcohol resistant bacillus (BAAR) spur was negative.

**Figure 1:**
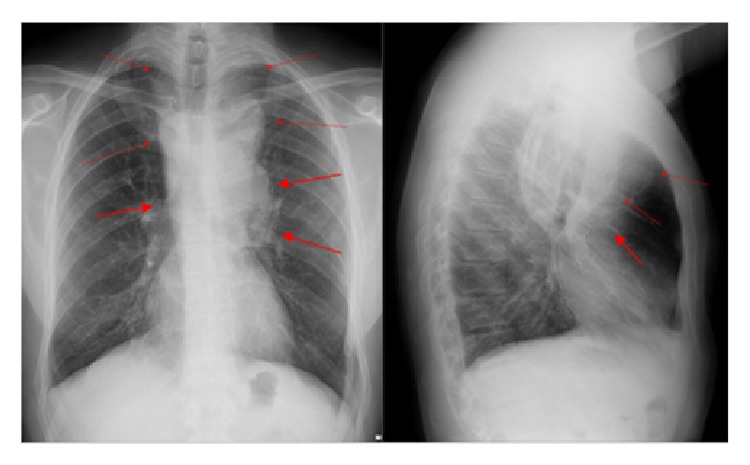
Chest X-ray in PA and Profile shows enlargement of the mediastinum (red arrows).

**Figure 2:**
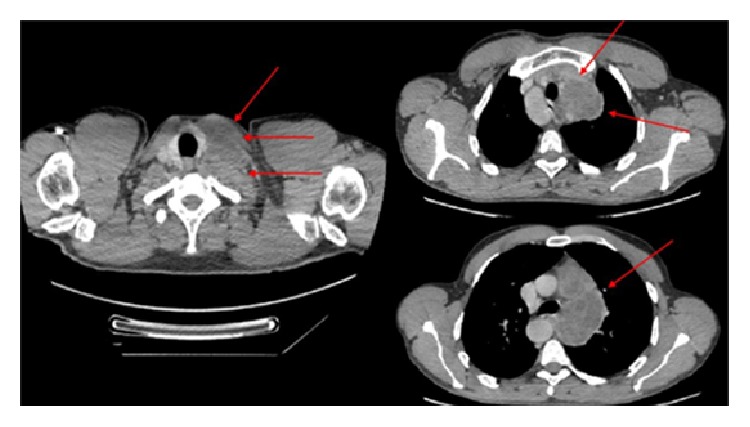
Chest tomography in axial section showing cervical and supraclavicular lymph nodes enlargement on the left. Left mediastinal masses with necrotic appearance (red arrows).

**Figure 3:**
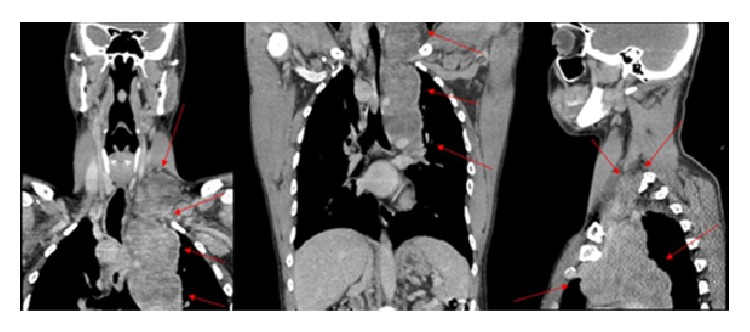
Chest tomography in coronal and sagittal sections demonstrating enlargement of the cervical and supraclavicular lymph node in the left. It also shows mediastinal masses and their extension (red arrows).

**Figure 4:**
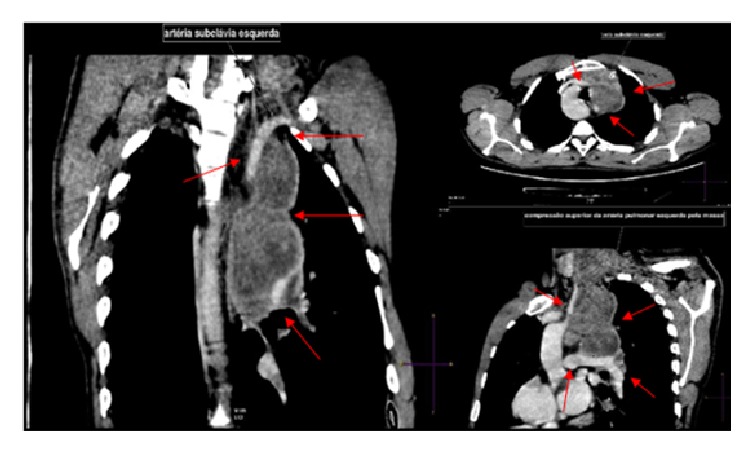
Chest tomography in axial, coronal and sagittal sections demonstrating the mediastinal masses and their relations with some mediastinal vessels (white and red arrows).

**Figure 5:**
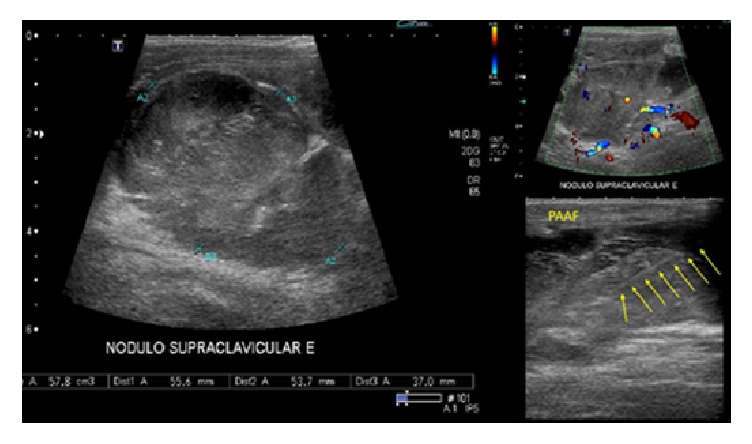
Ultrasonography of the neck demonstrating the shape and vascularization of left supraclavicular lymphadenopathy. It also demonstrates the puncture/biopsy by fine needle of the nodule (FNAB), highlighted by the yellow arrows.

He also performed same imaging studies such as chest X-ray (Figure 1), chest computed tomography (Figures 2, 3, and 4), and ultrasonography (Figure 5), which demonstrate mediastinal and left supraclavicular masses, interpreted as lymph node conglomerates of unknown etiology. Therefore the main diagnostics hypothesis was lymphoproliferative or granulomatous infectious diseases, especially tuberculosis.

**Figure 6:**
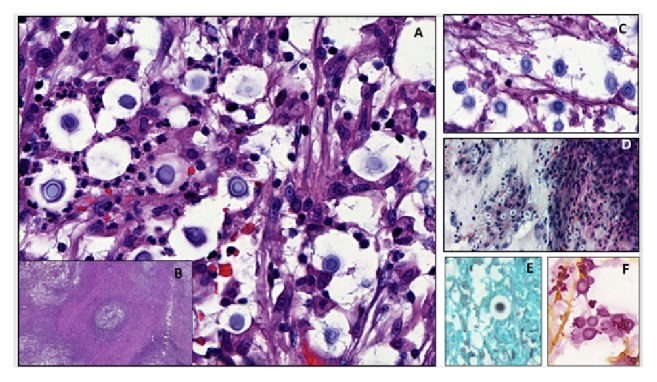
Microscopy slides: (A) Hematoxylin and eosin staining (H & E) observing spherical to oval yeasts with size variability. (B) Granulomas with peripheral fibrosis showing the chronicity of the process. (C) Larger increase in the center of the granuloma by identifying numerous yeasts with thick capsules of mucopolysaccharide giving the characteristic appearance of having a free space around them. (D) FNA of supraclavicular lymph node showing multiple spherical yeast structures. (E) Grocott-methenamine silver (GMS) positive staining highlighting the wall of the fungus. (F) Positive mucicarmine staining by radiating the fungus capsule.

**Figure 7:**
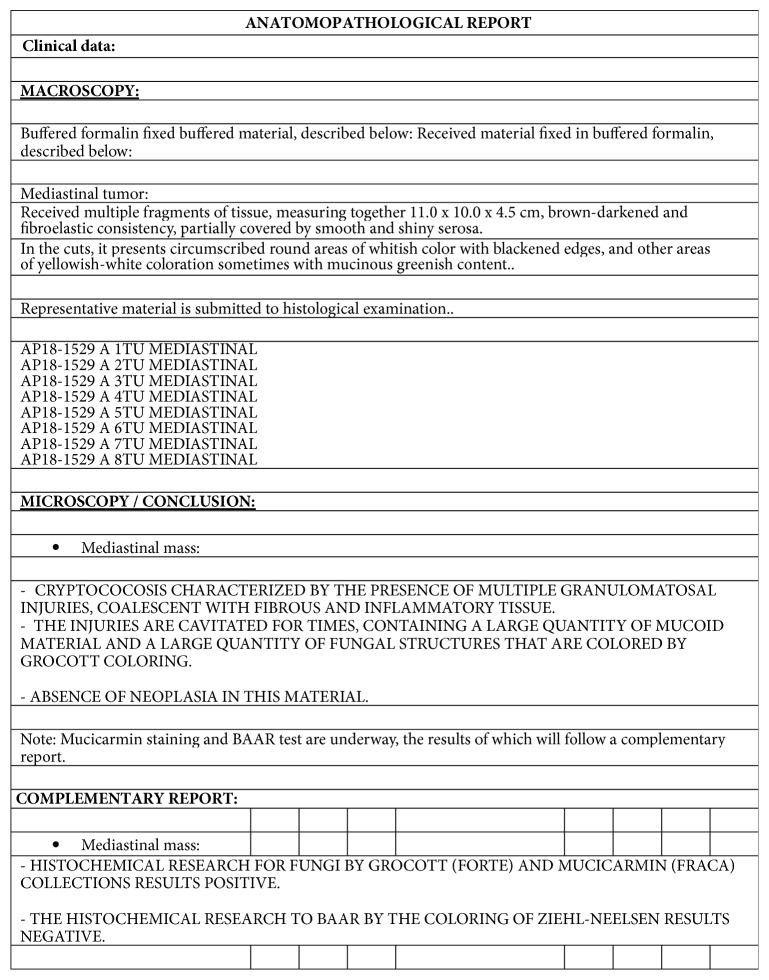
Histopathological description.

He underwent a fine needle aspiration (Figure 5) of the left supraclavicular mass. Histopathology (Figures 6 and 7) showed a granulomatous inflammation with fungal identification, and immunohistochemistry was positive for Grocott-methenamine silver nitrate and mucicarmine (Figures 6 and 7). The final diagnostic was* Cryptococcus neoformans* var.* gattii*. Ziehl-Neelsen coloration was negative (Figures 6 and 7).

He was first treated clinically, with intravenous antifungal therapy for almost 60 days (6 days of fluconazol being replaced with 57 days of flucytosine and 59 days of B-amphotericin lipidic complex), but he did not improve, with remaining pulmonary symptoms.

**Figure 8:**
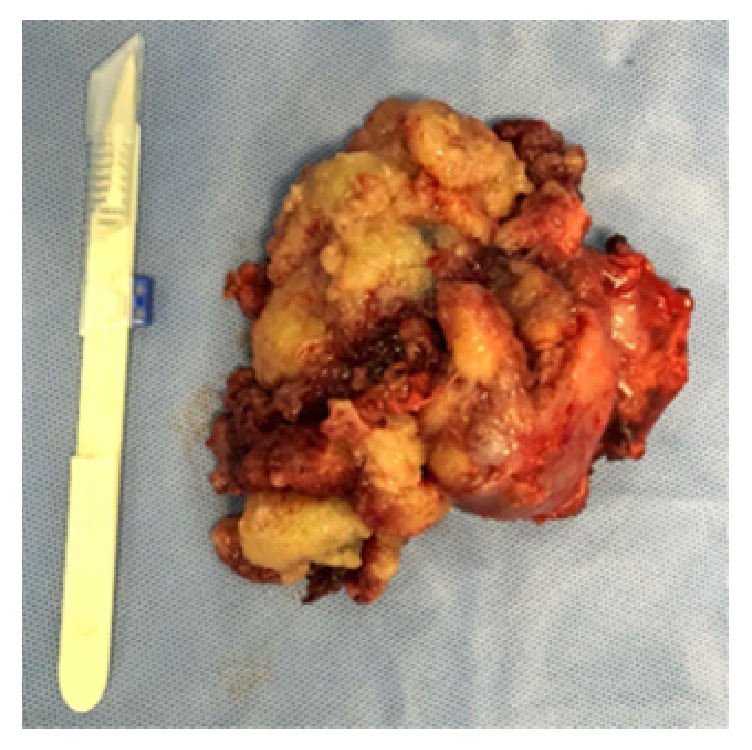
Surgical resection product.

**Figure 9:**
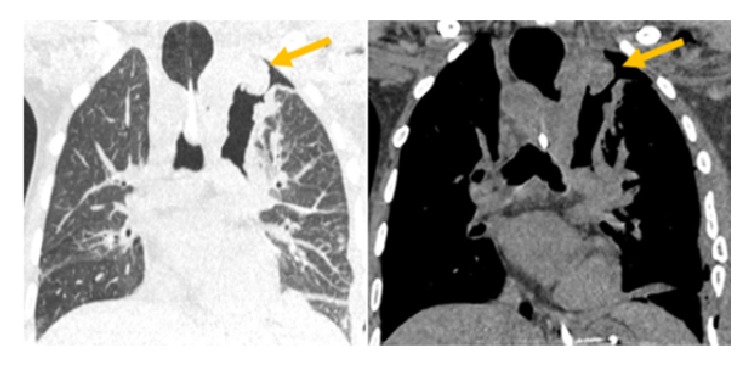
Chest tomography in coronal sections, in the lung and mediastinal windows, demonstrating the area of surgical manipulation and small residual lesion (yellow arrows).

Therefore, a multidisciplinary team decided for surgical resection. The lesion was almost entirely resected (Figures 8 and 9). Histopathology and cultures confirmed the lesion as cryptococcoma.

The patient was being followed clinically and radiologically (Figure 9) and had no symptoms or complications so far.

## 3. Discussion

*Cryptococcus* is a basidiomycetous yeast ubiquitous in the environment, but a major human fungal pathogen.* C*.* neoformans* and* C*.* gattii* are the two medically important species, particularly C.* neoformans*. var.* grubii which *is the main causative agent for the majority of cases of cryptococcosis. These microorganisms are typically a threat to immunocompromised patients (e.g., HIV‐infected patients, patients with long‐term glucocorticoid therapy, or patients after organ transplantation), but a number of immunocompetent cases have also been described. [11–15, 17].

Cryptococcal infection develops after inhalation of fungal spores predominantly found in soil contaminated with pigeon excreta. The host's immune status determines the dissemination and clinical course of infection. In immunocompetent hosts, these microorganisms tend to be localized without dissemination, and pulmonary lesions mainly present as solitary or multiple nodules [12–15, 17]. Lymph node involvement is rare and is usually part of the disseminated disease or an immune reconstitution inflammatory syndrome in HIV‐infected individuals [12–15, 17].

Lymphadenopathy and pulmonary parenchymal infiltrates are the dominant radiographic manifestations in immunocompromised hosts. In immunocompetent patients, solitary or multiple pulmonary nodules are common, but lymph node involvement is uncommon. Massive mediastinal lymphadenopathy is very rare, although some cases have been reported [18–20].

The diagnosis is usually based on isolation of the fungus from cultured clinical specimens, but it requires several days and a large amount of samples. The detection of cryptococcal capsular antigen in serum and CSF specimens by latex agglutination is one of the most helpful adjunct techniques to diagnose cryptococcosis because of its good sensitivity [11–15, 17].

Differential diagnosis is broad, including more frequent inflammatory and infectious diseases, such as primary tuberculosis, as well as neoplastic diseases, such as lymphoma. Cattleman's and neurogenic tumors are less frequent but should be included in atypical cases. It should be remembered that patients with Cushing's disease with hypercortisolism present a higher risk of opportunistic infections, among which is cryptococcosis [21].

As seen in the current case report, immunocompetent patient can also develop* Cryptococcus* mediastinal masses, though very rarely, the diagnosis of which being restricted to biopsy.

## 4. Conclusion

This case report is important for radiologists and medical community in general, as it demonstrates through imaging methods one infrequent spectrum of* Cryptococcus* infection, allowing clinical treatment (medication) previous to surgery.

Cryptococcosis has a wide range of presentations and some of them may simulate neoplastic disease. We must be attentive to atypical presentations of benign diseases, in order to offer effective treatment and avoid complications.
